# Supplementary material for: Methionine uptake via the SLC43A2 transporter is essential for regulatory T-cell survival
Source: Life Sci Alliance. 2022 Sep 9;5(12):e202201663. doi: 10.26508/lsa.202201663 (PMC9463494; doi:10.26508/lsa.202201663)

Fig 4C(i)

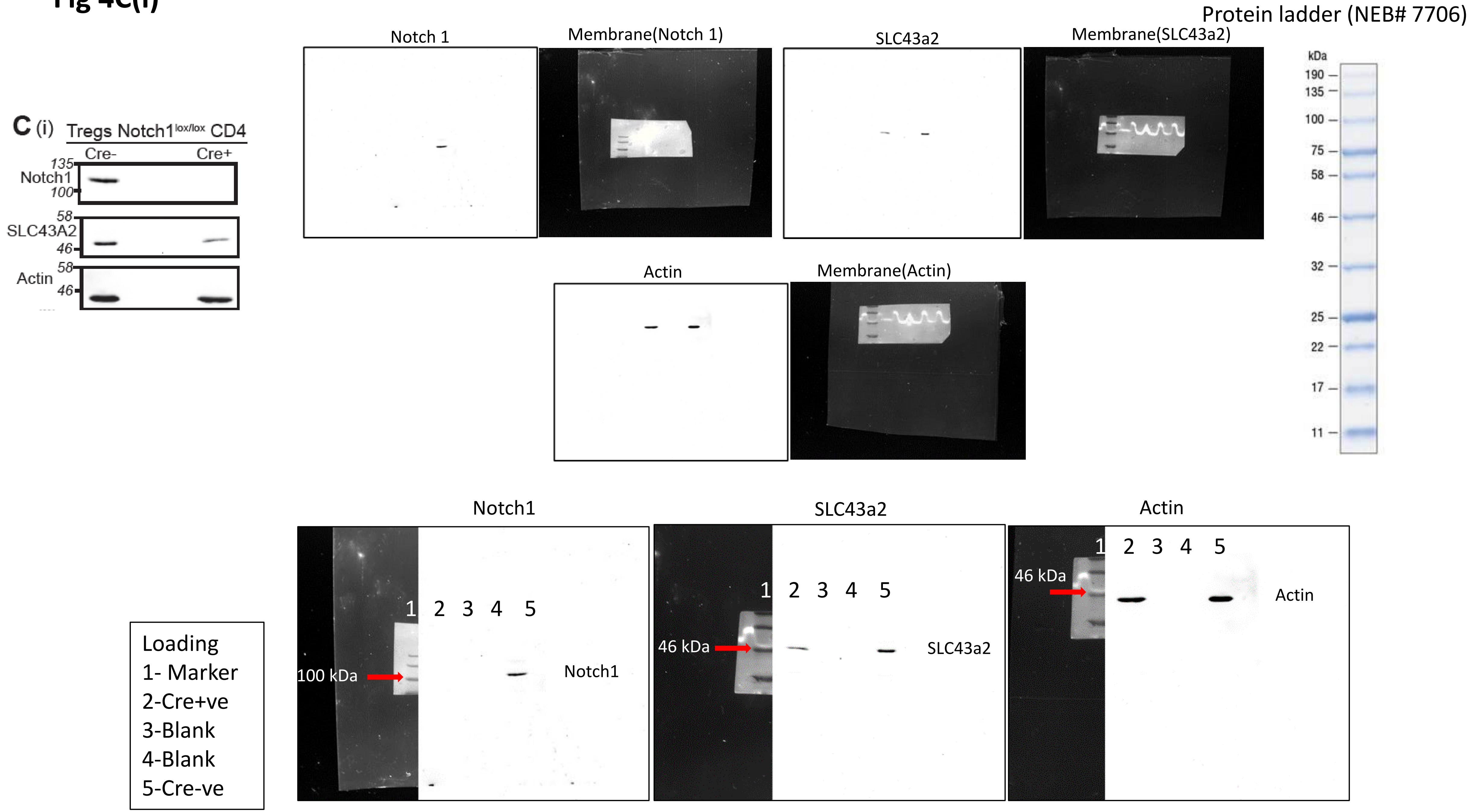
Protein ladder (NEB# 7706)
Notch 1
Membrane(Notch 1)
SLC43a2
Membrane(SLC43a2)
kDa
190
135
100
75
58
46
32
25
22
17
11
C (i) Tregs Notch1 lox/lox CD4
Cre-
Cre+
135
Notch1
100
58
SLC43A2
46
58
Actin
46
Actin
Membrane(Actin)
Notch1
SLC43a2
Actin
1 2 3 4 5
100 kDa
Notch1
46 kDa
SLC43a2
Actin
Loading
1- Marker
2-Cre+ve
3-Blank
4-Blank
5-Cre-ve

Fig 4Cii

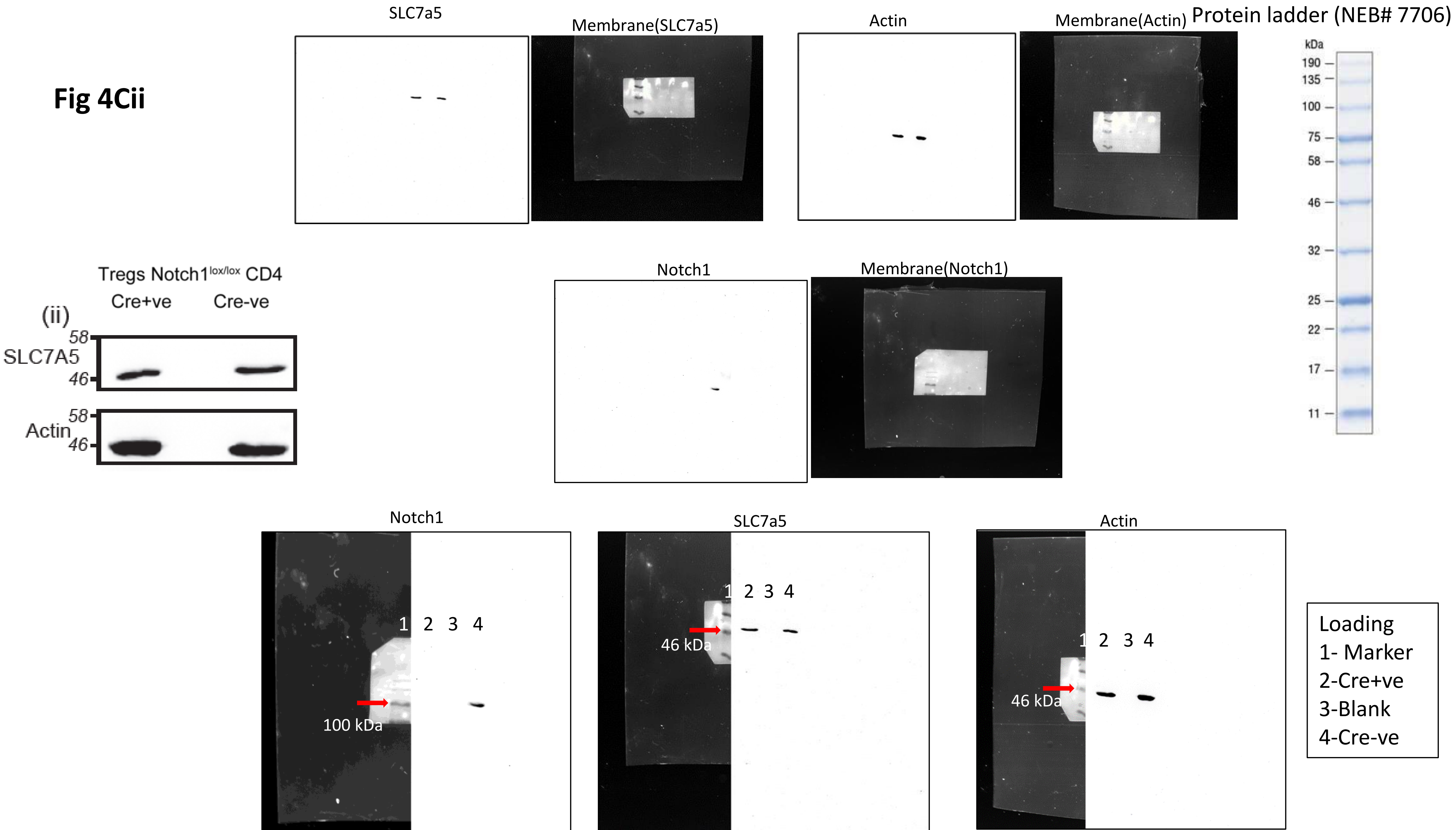
SLC7a5
Membrane(SLC7a5)
Actin
Membrane(Actin)
Protein ladder (NEB# 7706)
kDa
190
135
100
75
58
46
32
25
22
17
11
Tregs Notch1lox/lox CD4
Cre+ve
Cre-ve
(ii)
SLC7A5
58
46
Actin
58
46
Notch1
Membrane(Notch1)
Notch1
1 2 3 4
100 kDa
SLC7a5
1 2 3 4
46 kDa
Actin
1 2 3 4
46 kDa
Loading
1- Marker
2-Cre+ve
3-Blank
4-Cre-ve

Fig 4D

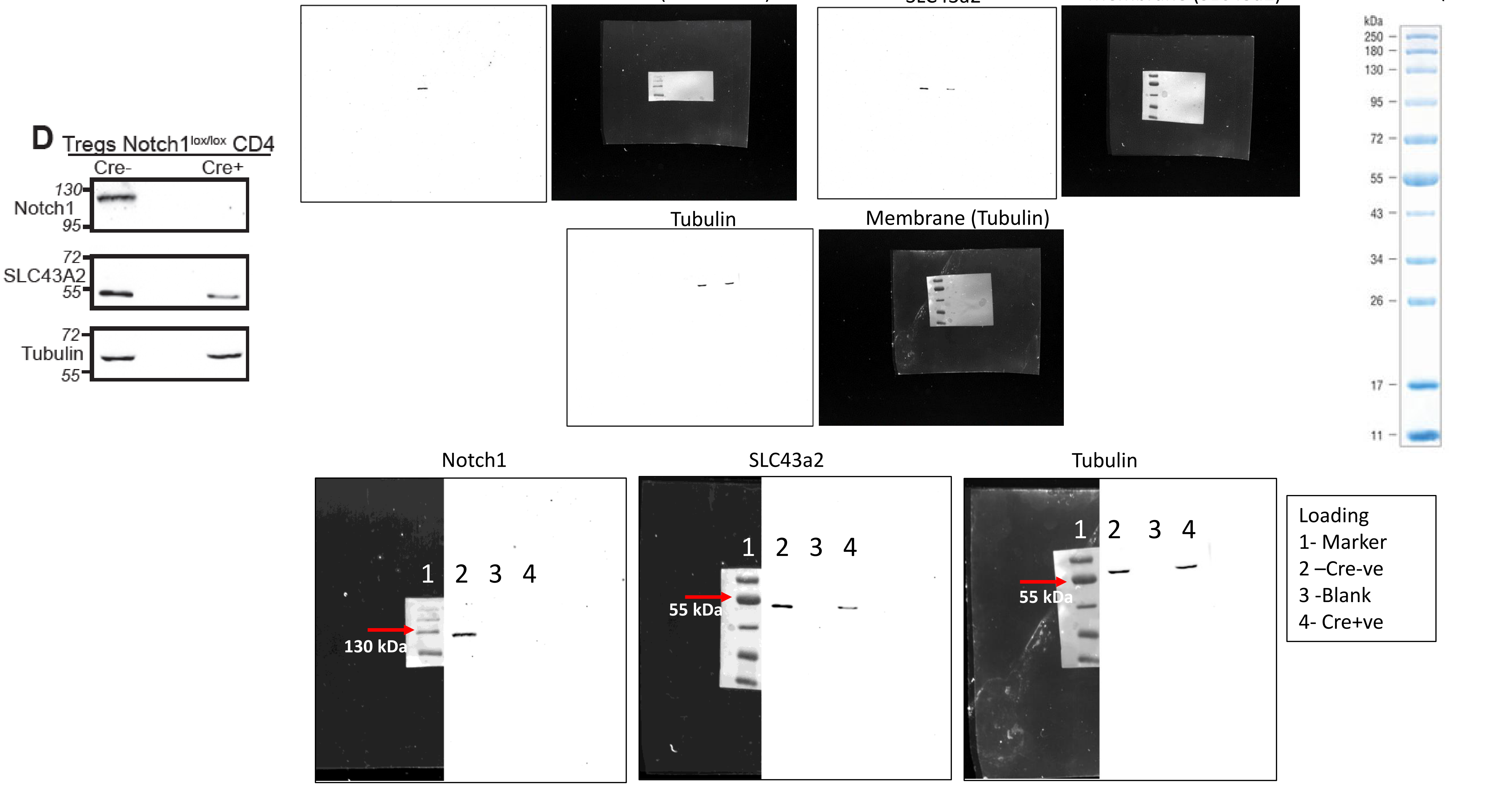

D Tregs Notch1lox/lox CD4
Cre-
Cre+
130
Notch1
95
72
SLC43A2
55
72
Tubulin
55
Notch1
Notch1(membrane)
SLC43a2
Membrane (SLC43a2)
Protein ladder (NEB#P7
kDa
250
180
130
95
72
55
43
34
26
17
11
Tubulin
Membrane (Tubulin)
Notch1
1 2 3 4
130 kDa
SLC43a2
1 2 3 4
55 kDa
Tubulin
1 2 3 4
55 kDa
Loading
1- Marker
2 –Cre-ve
3 -Blank
4- Cre+ve

Figure 4E

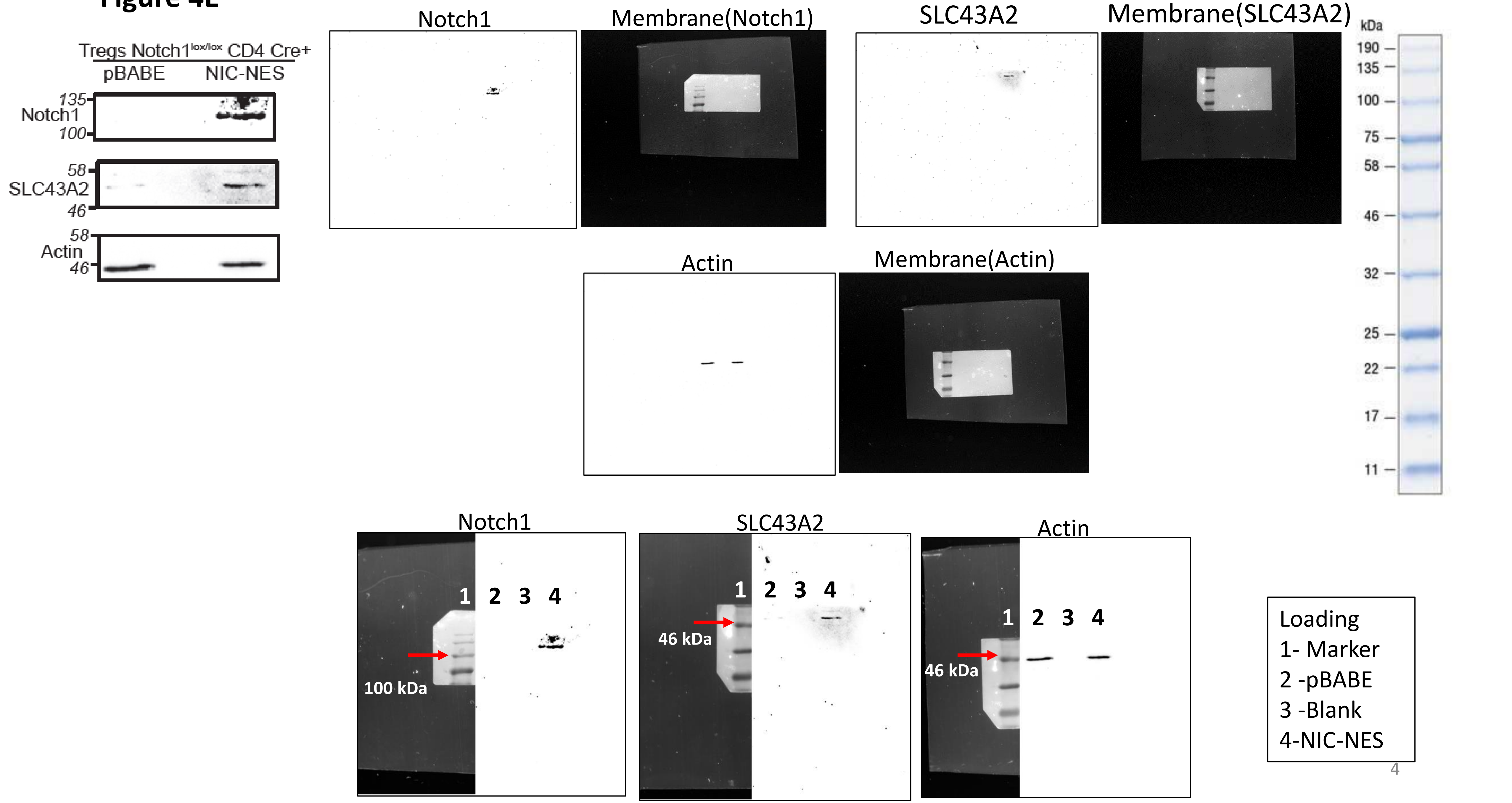
Tregs Notch1 lox/lox CD4 Cre+
pBABE
NIC-NES
135
Notch1
100
58
SLC43A2
46
58
Actin
46
Notch1
Membrane(Notch1)
SLC43A2
Membrane(SLC43A2)
Protein ladder (NEB# 7706)
kDa
190
135
100
75
58
46
32
25
22
17
11
Actin
Membrane(Actin)
Notch1
1 2 3 4
100 kDa
SLC43A2
1 2 3 4
46 kDa
Actin
1 2 3 4
46 kDa
Loading
1- Marker
2 -pBABE
3 -Blank
4-NIC-NES

Fig S3

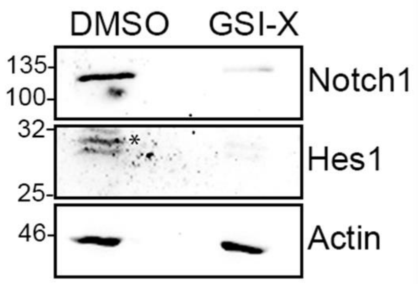

Protein ladder (NEB# 7706)

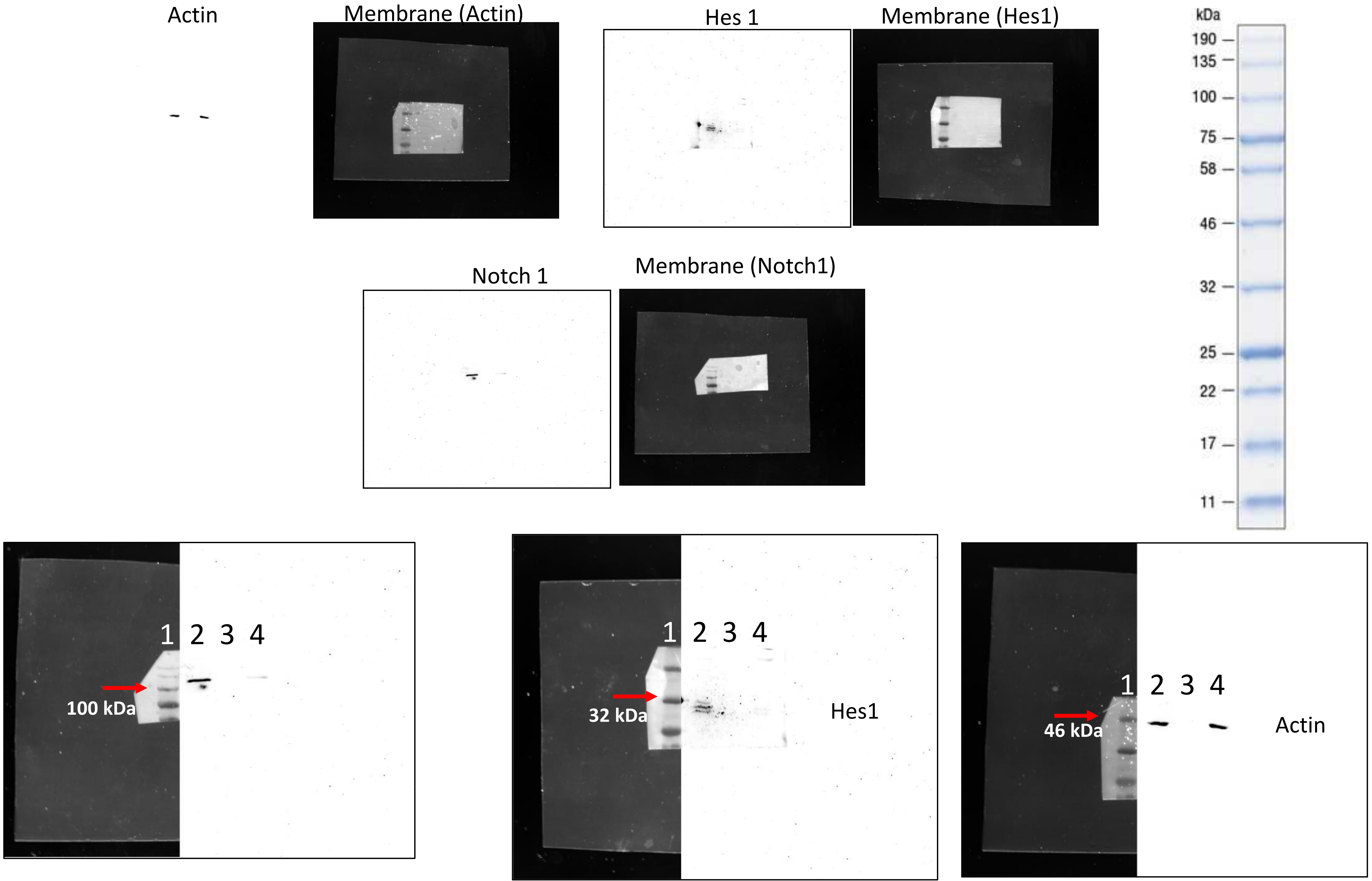

Loading

1- Marker
2 -DMSO
3 -Blank
4- GSI-X

Fig S4B

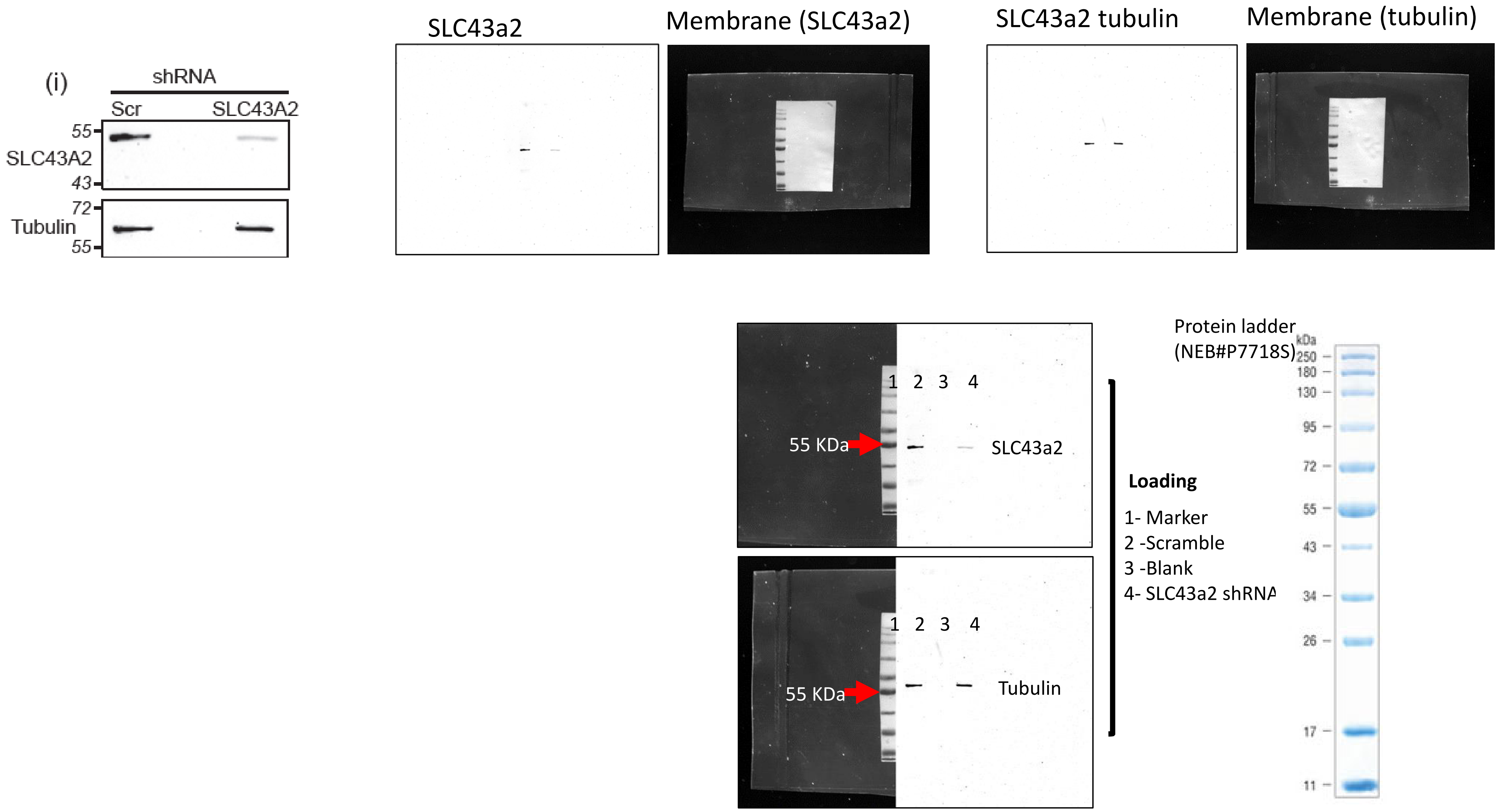
(i)
shRNA
Scr
SLC43A2
55
SLC43A2
43
72
Tubulin
55
SLC43a2
Membrane (SLC43a2)
SLC43a2 tubulin
Membrane (tubulin)
1 2 3 4
55 KDa
SLC43a2
1 2 3 4
55 KDa
Tubulin
Loading
1- Marker
2 -Scramble
3 -Blank
4- SLC43a2 shRNA
Protein ladder
(NEB#P7718S)
kDa
250
180
130
95
72
55
43
34
26
17
11

Fig S4C

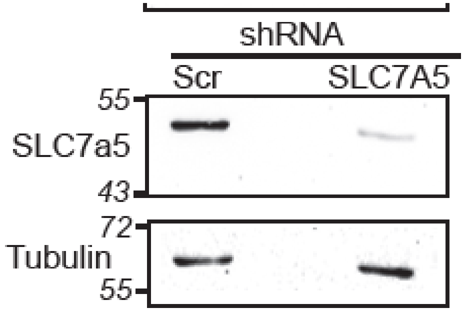

SLC7A5

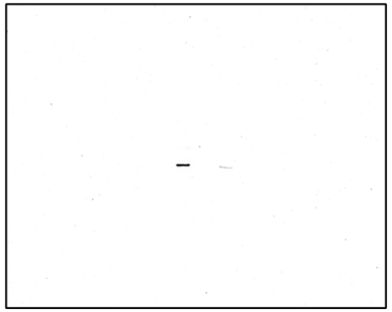

Membrane (SLC7A5)

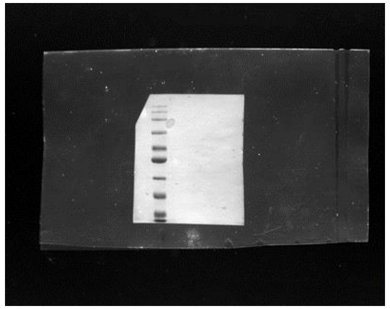

SLC7A5 tubulin

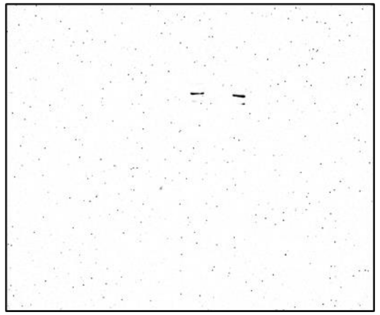

Membrane(tubulin)

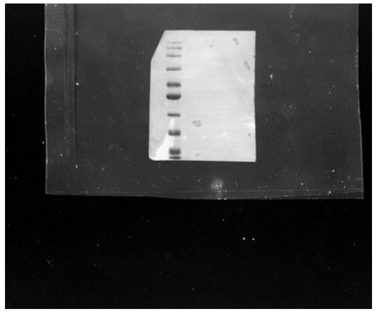

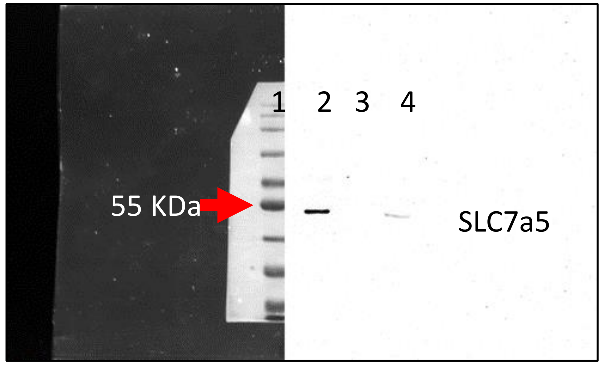

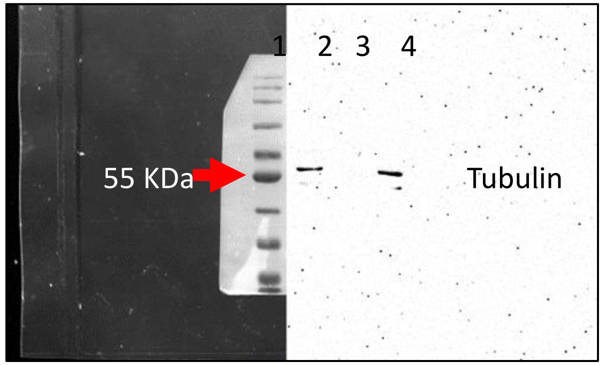

Loading

1- Marker
2 -Scramble
3 -Blank
4- SLC7a5 shRNA

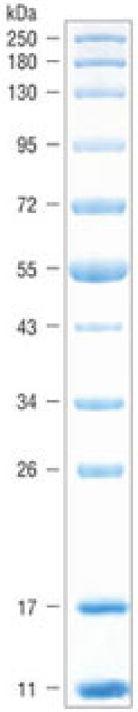

Fig S4D

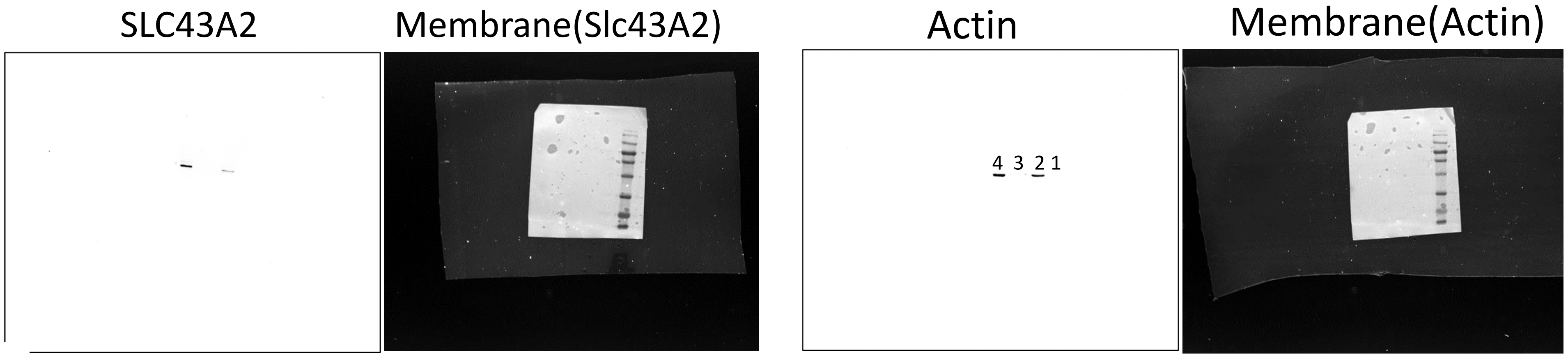
SLC43A2
Membrane(Slc43A2)
Actin
4 3 2 1
Membrane(Actin)

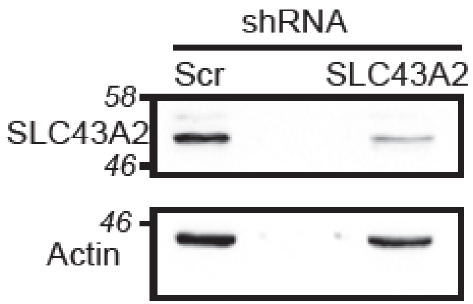
shRNA
Scr
SLC43A2
58
SLC43A2
46
46
Actin

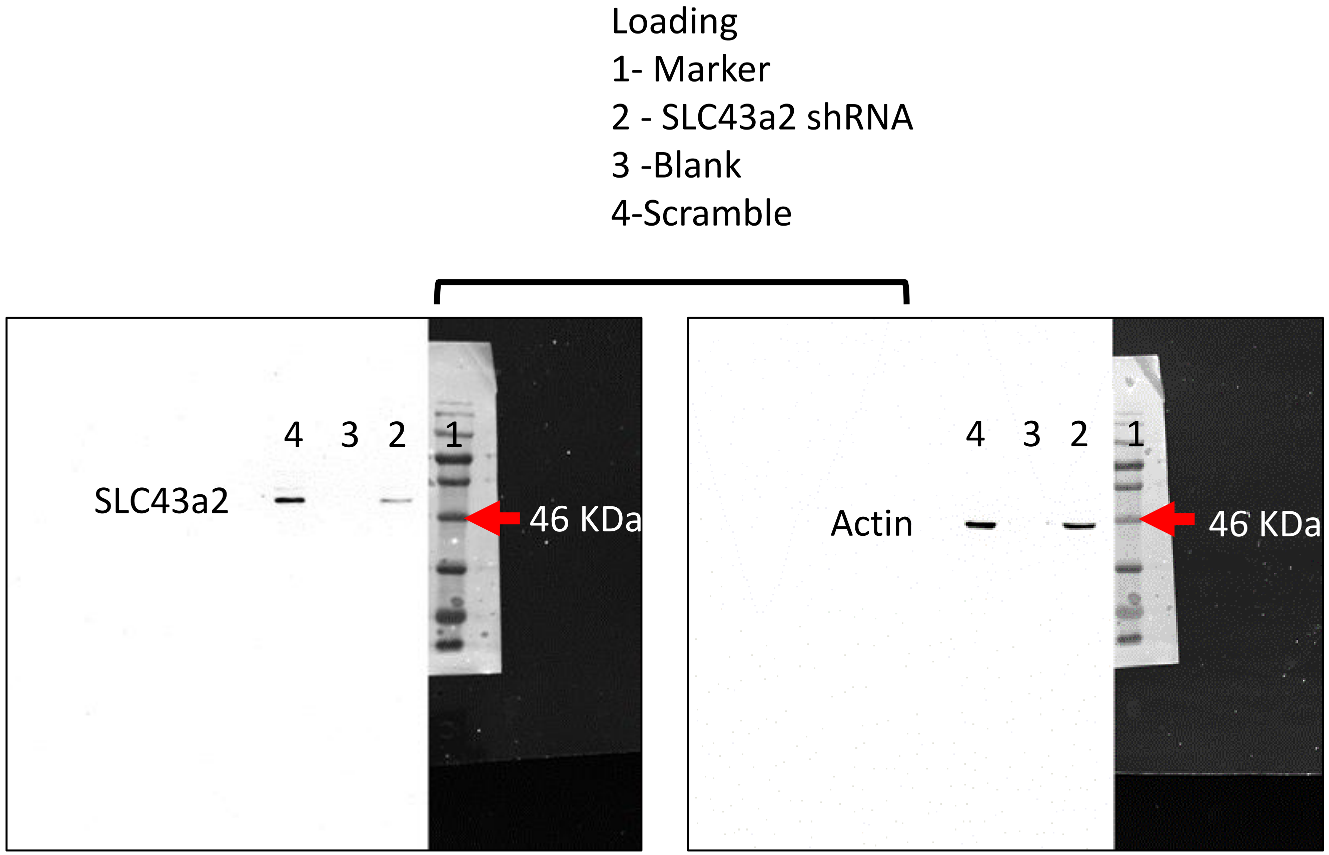
Loading
1- Marker
2 - SLC43a2 shRNA
3 -Blank
4-Scramble
SLC43a2
4 3 2 1
46 KDa
Actin
4 3 2 1
46 KDa

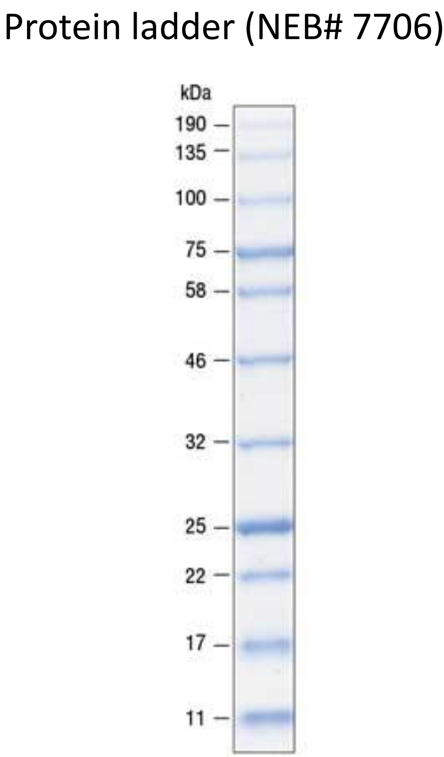
Protein ladder (NEB# 7706)
kDa
190
135
100
75
58
46
32
25
22
17
11

FigS5A

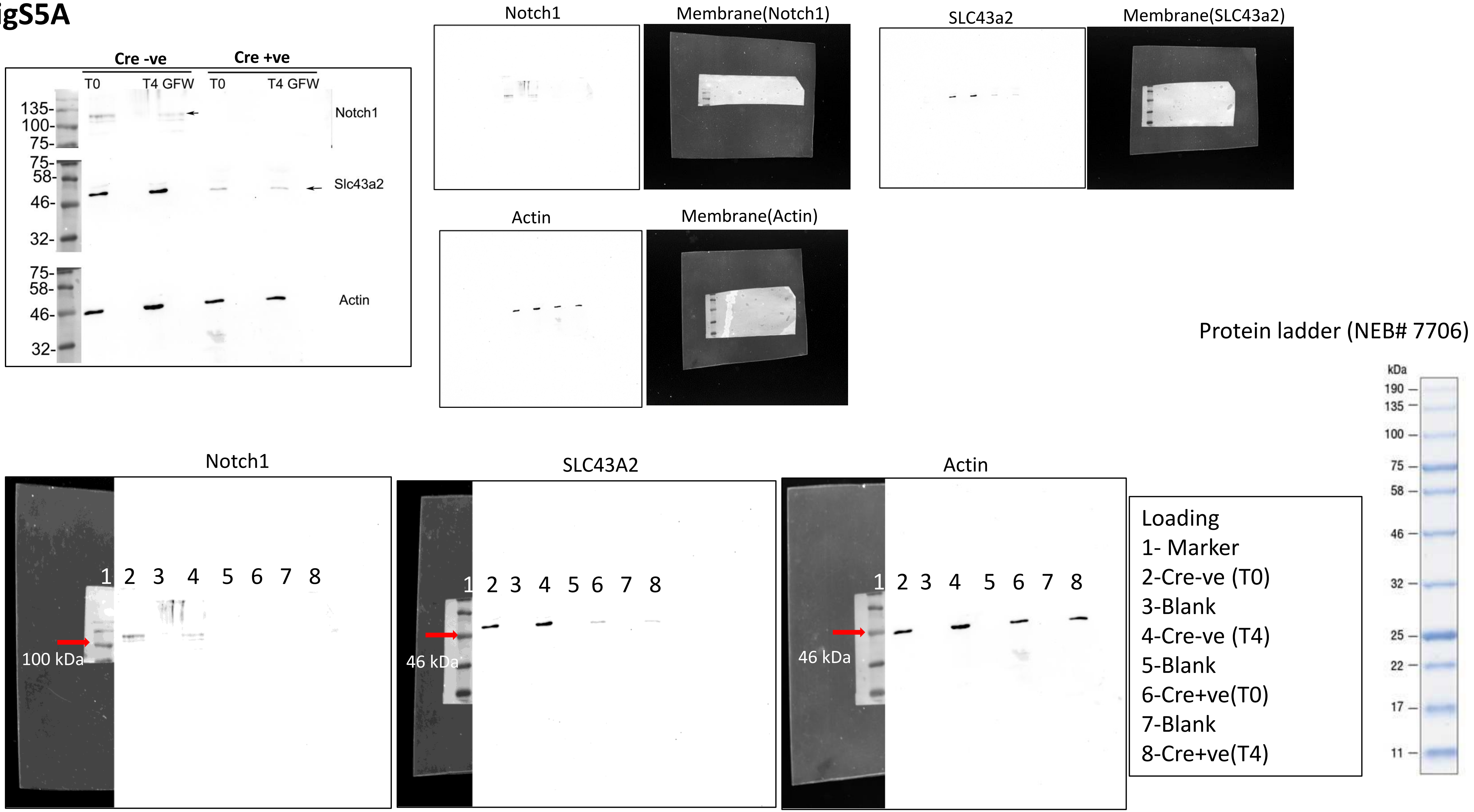

Cre -ve
Cre +ve
T0
T4 GFW
T0
T4 GFW
135-
100-
75-
Notch1
75-
58-
46-
32-
Slc43a2
75-
58-
46-
32-
Actin
Notch1
Membrane(Notch1)
SLC43a2
Membrane(SLC43a2)
Actin
Membrane(Actin)
Protein ladder (NEB# 7706)
kDa
190
135
100
75
58
46
32
25
22
17
11
Notch1
1 2 3 4 5 6 7 8
100 kDa
SLC43A2
1 2 3 4 5 6 7 8
46 kDa
Actin
1 2 3 4 5 6 7 8
46 kDa
Loading
1- Marker
2-Cre-ve (T0)
3-Blank
4-Cre-ve (T4)
5-Blank
6-Cre+ve(T0)
7-Blank
8-Cre+ve(T4)

Fig S5B

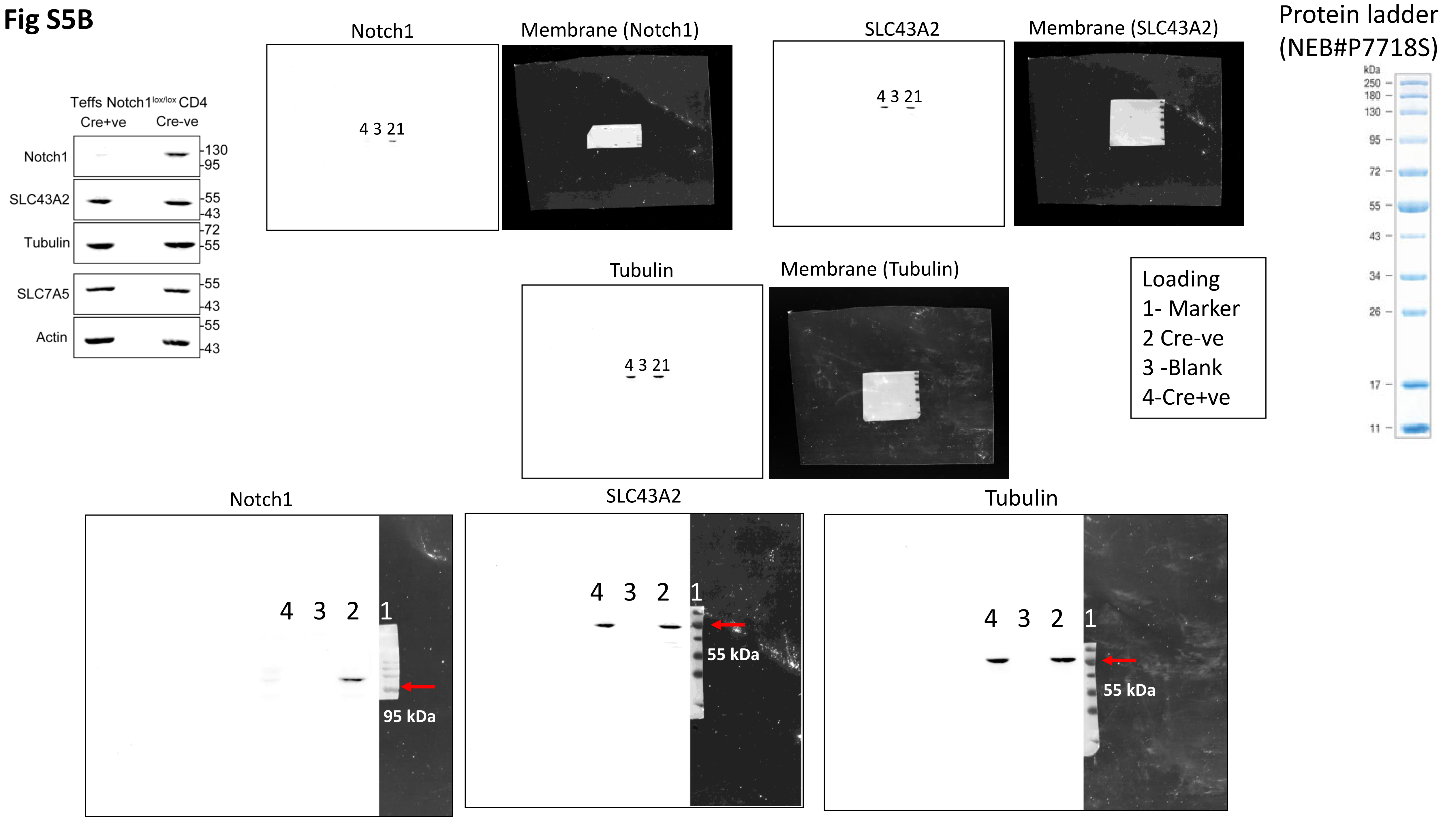
Teffs Notch1 lox/lox CD4
Cre+ve
Cre-ve
Notch1
SLC43A2
Tubulin
SLC7A5
Actin
130
95
55
43
72
Membrane (Notch1)
Membrane (SLC43A2)
Membrane (Tubulin)
4 3 2 1
Protein ladder (NEB#P7718S)
kDa
250
180
130
95
72
55
43
34
26
17
11
Loading
1- Marker
2 Cre-ve
3 -Blank
4-Cre+ve
95 kDa
55 kDa

Fig. S5Ci

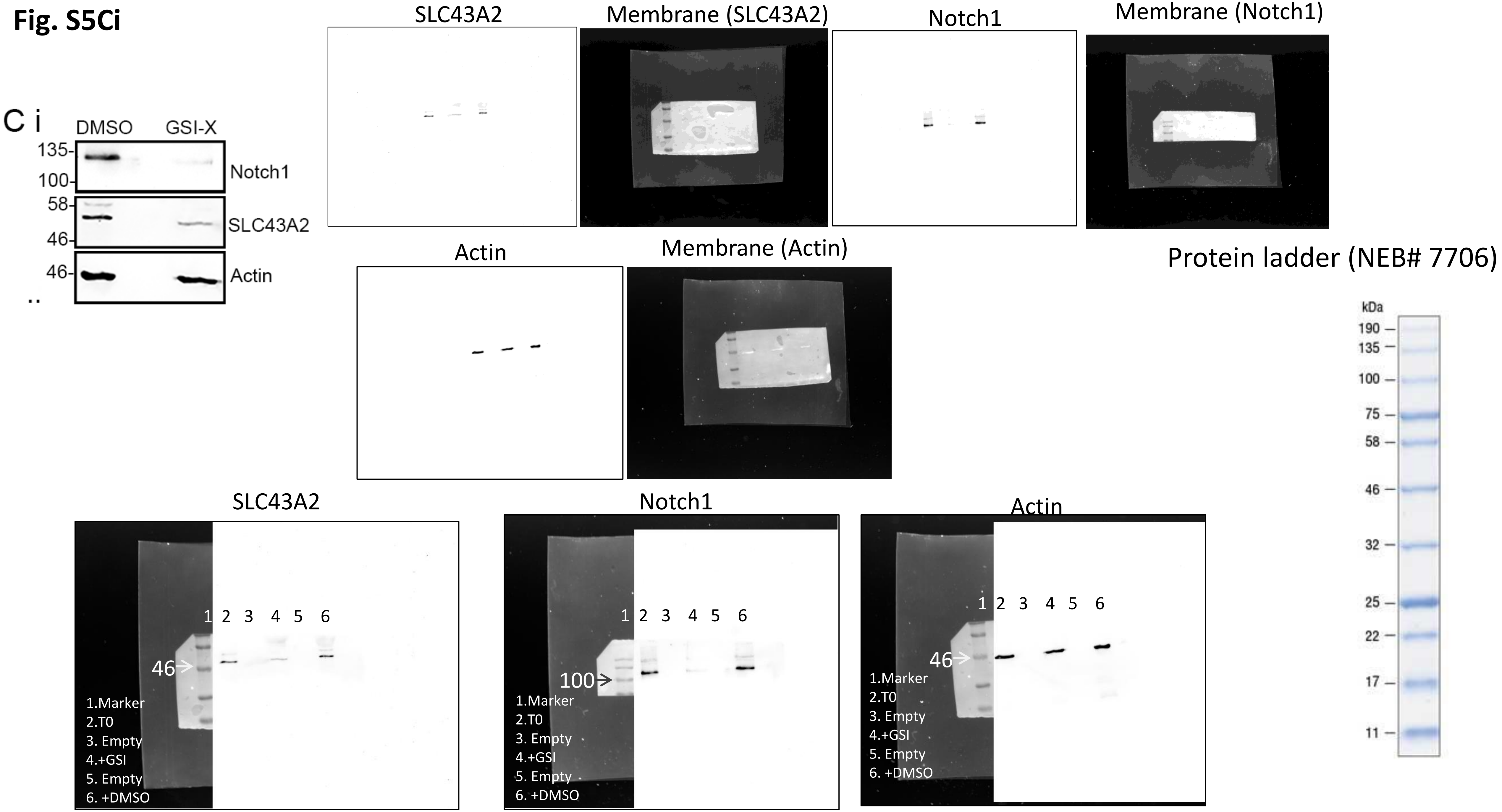
C i
DMSO
GSI-X
135-
100-
Notch1
58-
46-
SLC43A2
46-
Actin
SLC43A2
Membrane (SLC43A2)
Notch1
Membrane (Notch1)
Actin
Membrane (Actin)
Protein ladder (NEB# 7706)
kDa
190
135
100
75
58
46
32
25
22
17
11
SLC43A2
1 2 3 4 5 6
46
1.Marker
2.T0
3. Empty
4.+GSI
5. Empty
6. +DMSO
Notch1
1 2 3 4 5 6
100
1.Marker
2.T0
3. Empty
4.+GSI
5. Empty
6. +DMSO
Actin
1 2 3 4 5 6
46
1.Marker
2.T0
3. Empty
4.+GSI
5. Empty
6. +DMSO

Fig. S5Cii

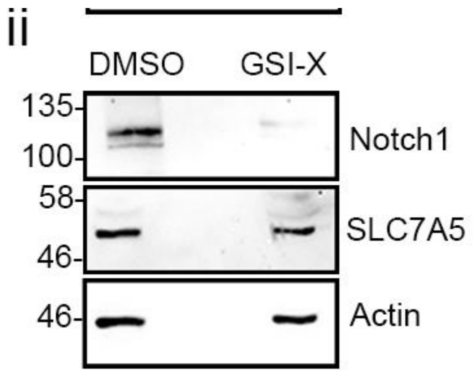

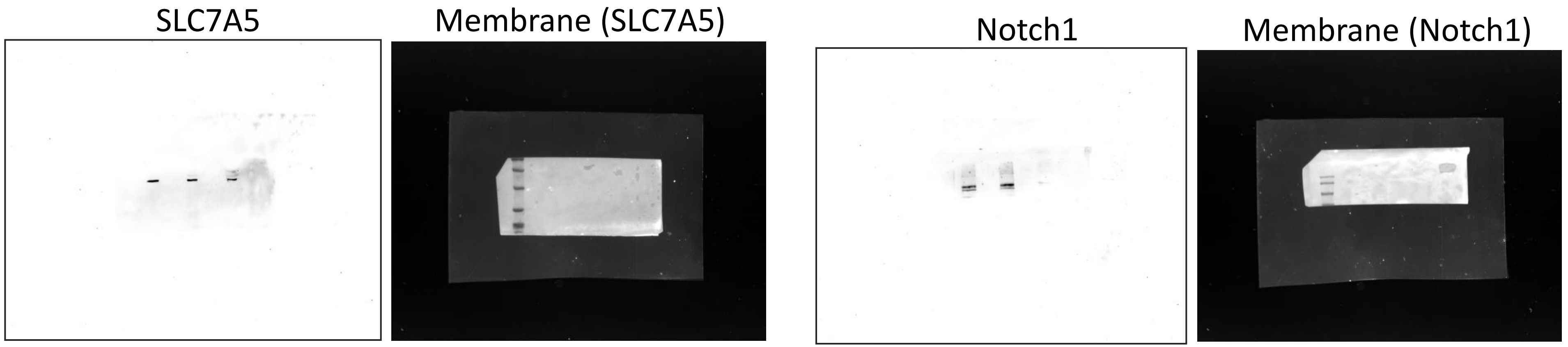

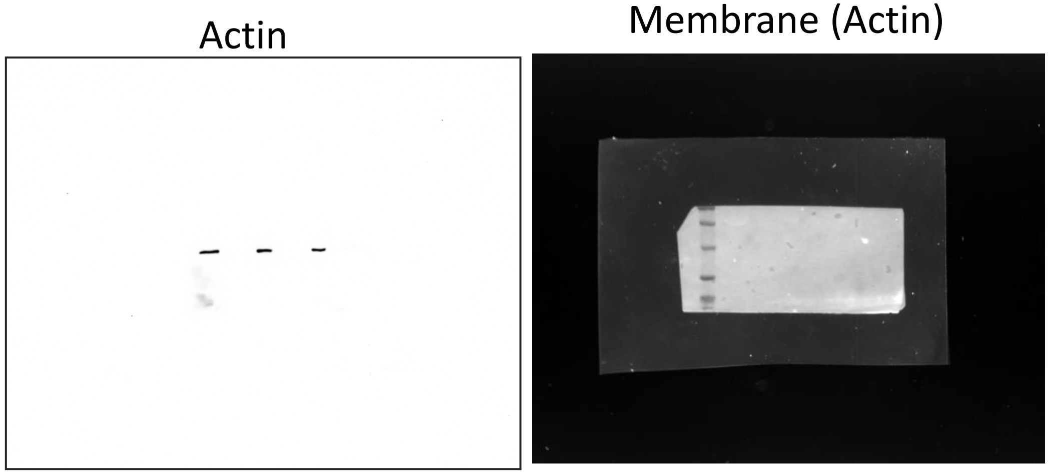

Protein ladder (NEB# 7706)

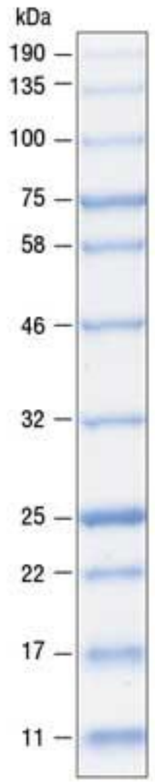

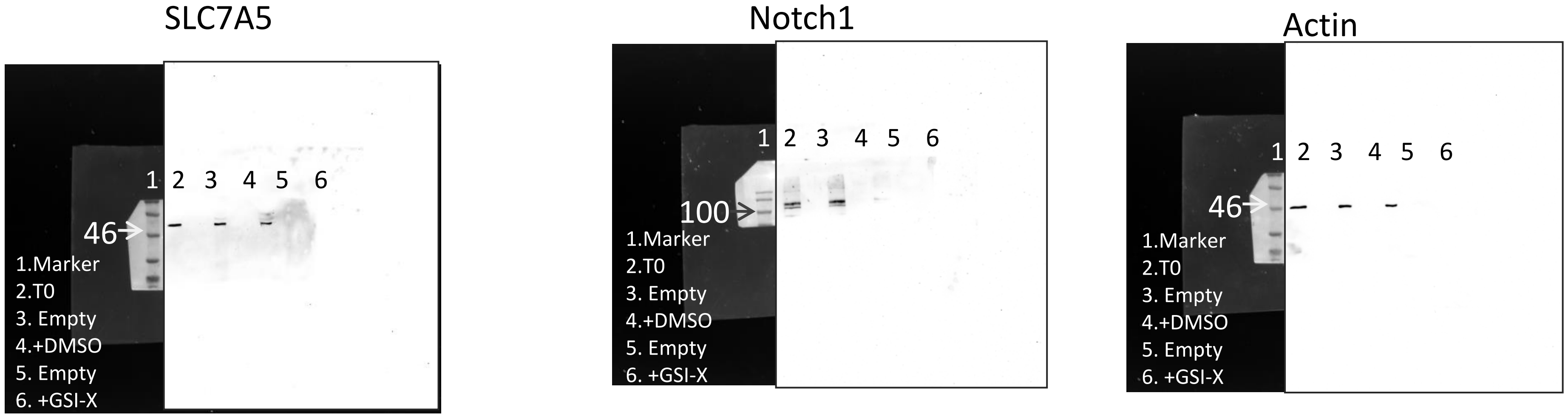

Fig S5D

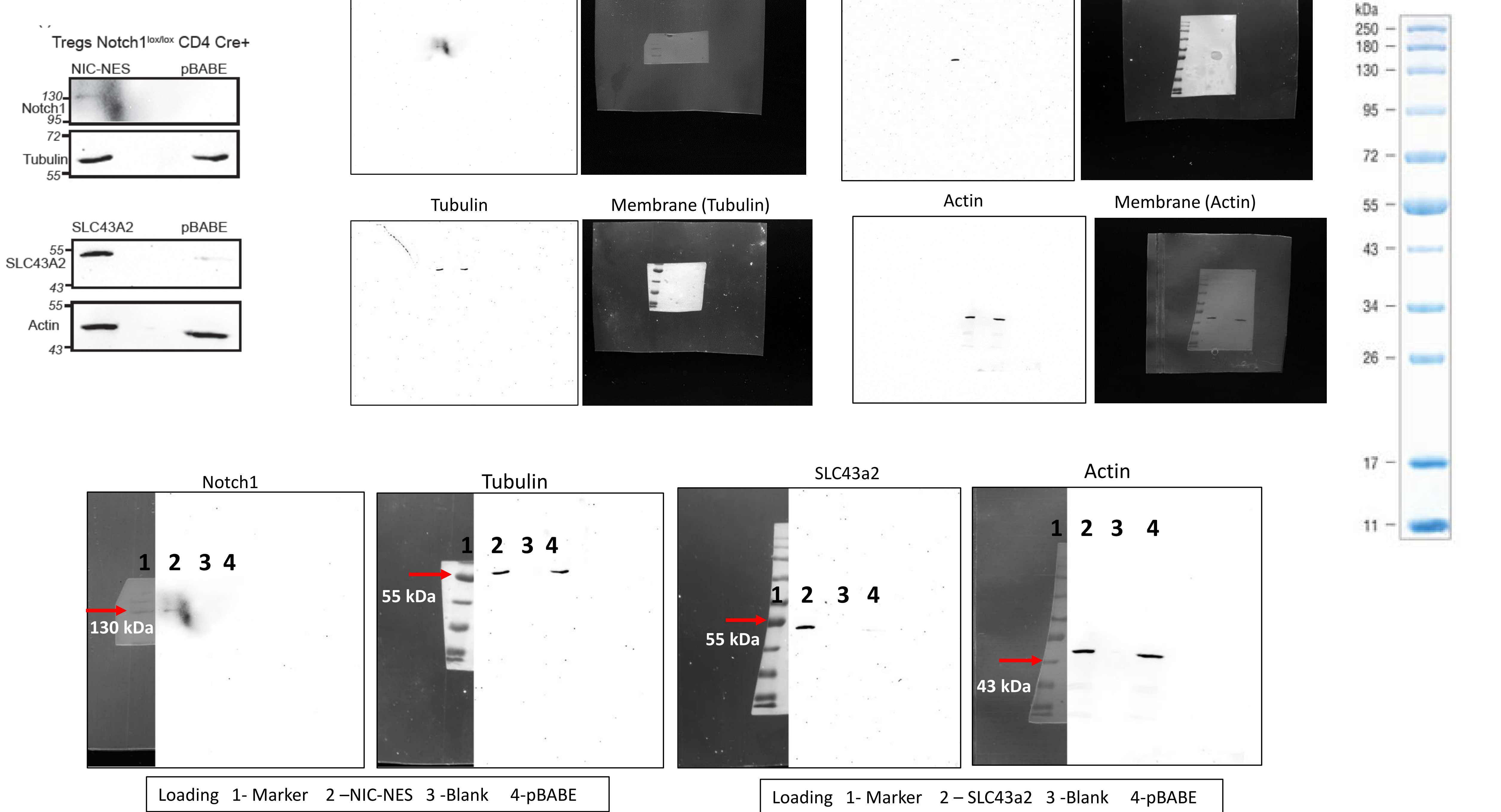

Tregs Notch1lox/lox CD4 Cre+
NIC-NES
pBABE
130
Notch1
95
72
Tubulin
55
SLC43A2
pBABE
55
SLC43A2
43
55
Actin
43
Notch1
Membrane (Notch1)
SLC43a2
Membrane (SLC43a2)
Protein ladder (NEB#P7718S)
kDa
250
180
130
95
72
55
43
34
26
17
11
Tubulin
Membrane (Tubulin)
Actin
Membrane (Actin)
Notch1
1 2 3 4
130 kDa
Tubulin
1 2 3 4
55 kDa
SLC43a2
1 2 3 4
55 kDa
Actin
1 2 3 4
43 kDa
Loading 1- Marker 2 –NIC-NES 3 -Blank 4-pBABE
Loading 1- Marker 2 – SLC43a2 3 -Blank 4-pBABE

Fig S2

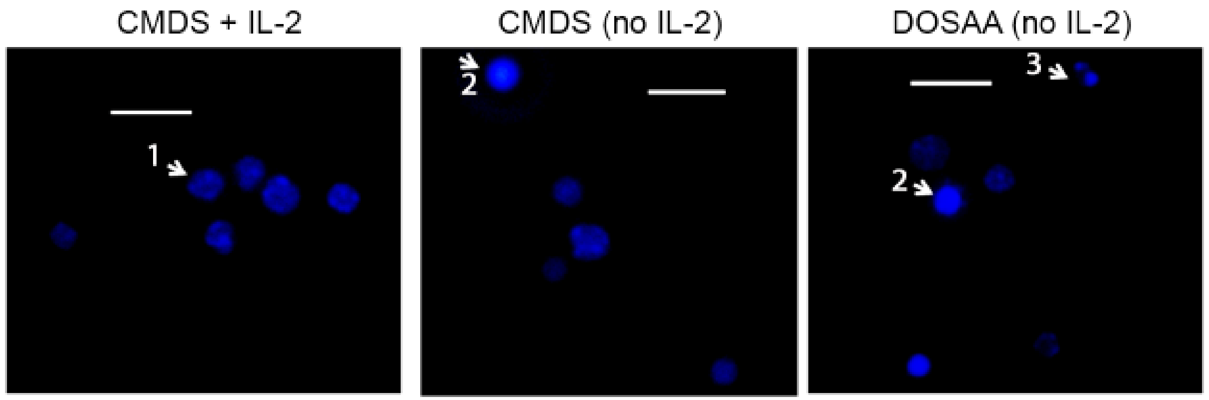

Arrowhead shows dead cell
Scale bar: 10µm

1. Live nucleus
2. Chromatin condensation
3. Nuclear fragmentation

Apoptotic nuclei (2, 3)

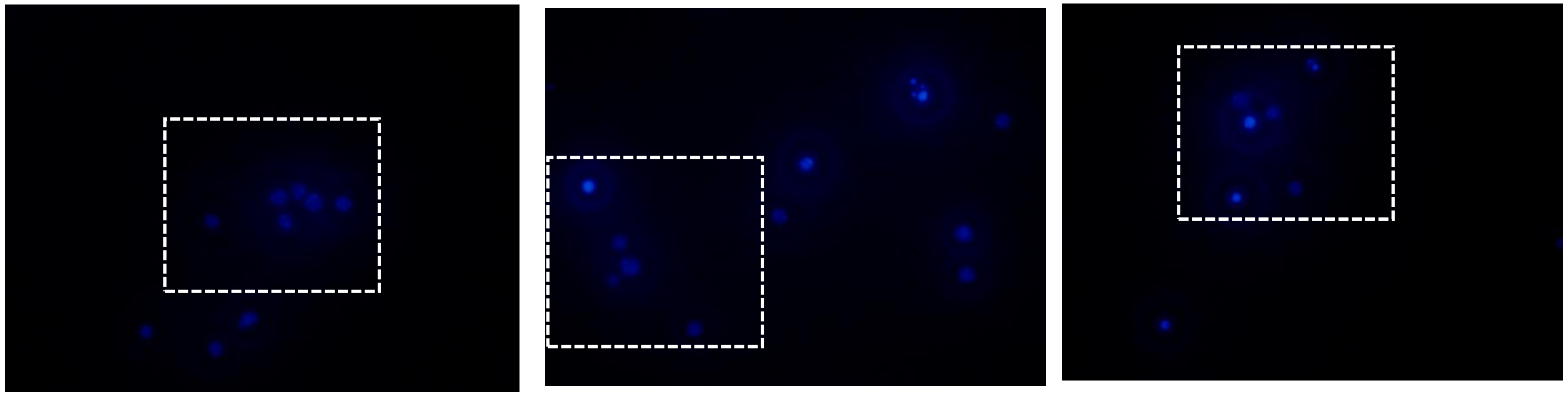

CMDS IL2

CMDS_3

DoSAA_4

CMDS = CMDS no IL-2

DOSAA= DOSAA no IL-2

Fig S4E

E i

Scr SLC43A2

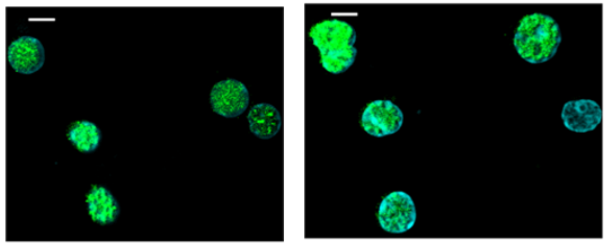

Foxp3 (green) Nucleus (blue)

ii

Percent Foxp3+ cells		
	n=1	n=2
Scrambled	91	85
Slc43a2	89	84

Scale bar: 5µm

Scr SLC43A2

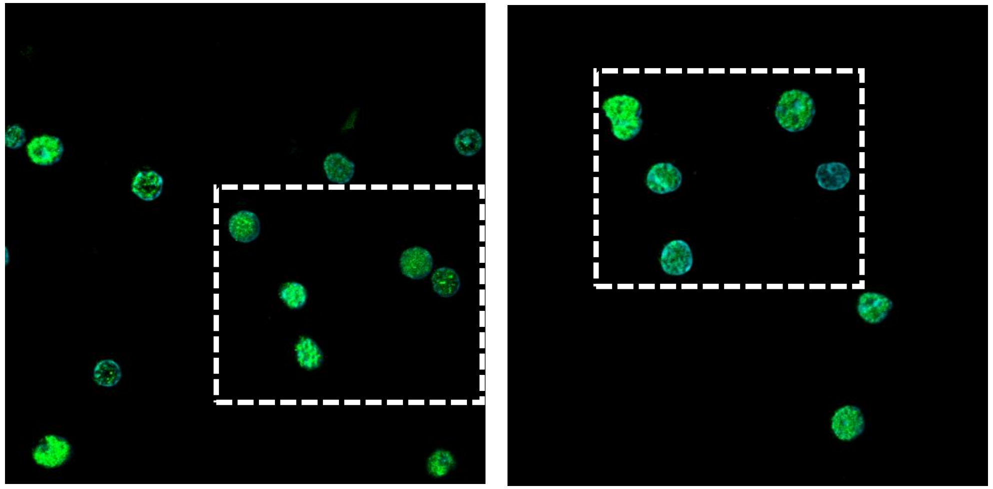

Fig 2D

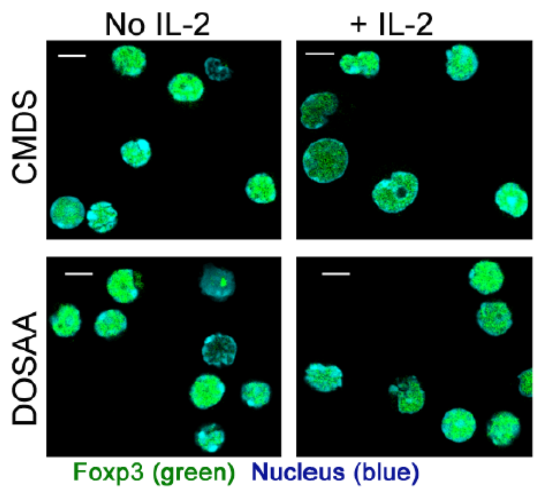

Percent Foxp3+ cells		
	n=1	n=2
CMDS no IL-2	87.6	87.7
CMDS + IL-2	80.4	85.0
DOSAA no IL-2	81.6	79.8
DOSAA + IL-2	73.7	76.1

Scale bar: 5µm

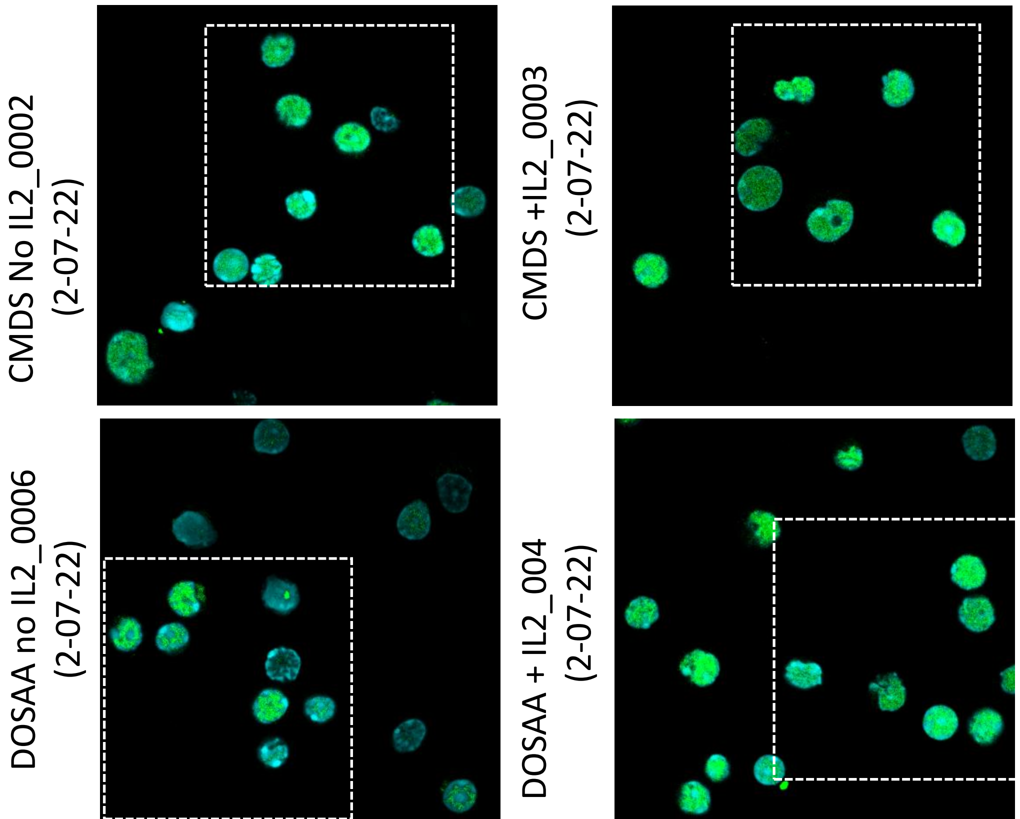

Supplement: Supplementary file 2 [file LSA-2022-01663_SdataF4_FS3_FS4_FS5_FS2_F2.pdf]
