# Supplementary material for: Methionine uptake via the SLC43A2 transporter is essential for regulatory T-cell survival
Source: Life Sci Alliance. 2022 Sep 9;5(12):e202201663. doi: 10.26508/lsa.202201663 (PMC9463494; doi:10.26508/lsa.202201663)
Supplement: Supplementary file 3 [file LSA-2022-01663_TableS1.docx]

**Table SI**

| **Primers used for sub cloning SLC43A2 into pBABE vector.** | | |
| --- | --- | --- |
| **Gene** | **Forward (5’–3’)** | **Reverse (5’–3’)** |
| SLC43A2 | BamH1-CGCGGATCCATGGCGCCCACC | EcoR1- CCGGAATTCTTACACGAAGGCCTCCT |
| **Primers used for RT PCR against murine gene** | | |
| HPRT | TCAGTCAACGGGGGACATAAA | GGGGCTGTACTGCTTAACCAG |
| Actin | TGGGTCAGAAGGACTCCTATG | CAGGCAGCTCATAGCTCTTCT |
| Notch1 | ACAGTGCAACCCCCTGTATG | TCTAGGCCATCCCACTCACA |
| SLC3A2 | TGCTCAGGCTGACATTGTAGC | TCAGCCAAGTACAAGGGTGC |
| SLC43A1 | TTCACATGGTCTGGCCTGG | TGTGGTCCAAGGCTAACCC |
| SLC43A2 | ACAGTTTGGTAGCCTCACTGG | CCGGTAGCAGATGAGGTAAAGG |
| SLC6A17 | CCTTCATCAACTTCTTCACCTC | CGACCACACACTTCTCATTC |
| SLC7A5 | CTGGATCGAGCTGCTCATC | GTTCACAGCTGTGAGGAGC |
| SLC1A5 | TACATTCTGTGCTGCCTGCT | ATGAAACGGCTGATGTGC |
| SLC7A8 | CTAGCCTCCAATGCAGTTGC | GGCTCCAGCAAAGAACAGC |
| **Target shRNA sequence** | | |
| SLC43A2 shRNA | 3’-TTGTGCGAGGATTCATCCACTCTGCCGTA-5’ | |
| SLC7A5 shRNA | 3’-CGCCTACGGAGGATGGAACTATCTGAATT-5’ | |

**Table SI.** Primers and shRNA sequences used in the study.
